# Levels and correlates of non-adherence to WHO recommended inter-birth intervals in Rufiji, Tanzania

**DOI:** 10.1186/1471-2393-12-152

**Published:** 2012-12-13

**Authors:** Amon Exavery, Sigilbert Mrema, Amri Shamte, Kristin Bietsch, Dominic Mosha, Godfrey Mbaruku, Honorati Masanja

**Affiliations:** 1 fakara Health Institute (IHI), Plot 463, Kiko Avenue, off Old Bagamoyo Road, P.O. Box 78373, Dar es Salaam, Mikocheni, Tanzania; 2 Office of Population Research, Princeton University, Princeton, NJ, 08540, USA

## Abstract

**Background:**

Poorly spaced pregnancies have been documented worldwide to result in adverse maternal and child health outcomes. The World Health Organization (WHO) recommends a minimum inter-birth interval of 33 months between two consecutive live births in order to reduce the risk of adverse maternal and child health outcomes. However, birth spacing practices in many developing countries, including Tanzania, remain scantly addressed.

**Methods:**

Longitudinal data collected in the Rufiji Health and Demographic Surveillance System (HDSS) from January 1999 to December 2010 were analyzed to investigate birth spacing practices among women of childbearing age. The outcome variable, non-adherence to the minimum inter-birth interval, constituted all inter-birth intervals <33 months long. Inter-birth intervals ≥33 months long were considered to be adherent to the recommendation. Chi-Square was used as a test of association between non-adherence and each of the explanatory variables. Factors affecting non-adherence were identified using a multilevel logistic model. Data analysis was conducted using STATA (11) statistical software.

**Results:**

A total of 15,373 inter-birth intervals were recorded from 8,980 women aged 15–49 years in Rufiji district over the follow-up period of 11 years. The median inter-birth interval was 33.4 months. Of the 15,373 inter-birth intervals, 48.4% were below the WHO recommended minimum length of 33 months between two live births. Non-adherence was associated with younger maternal age, low maternal education, multiple births from the preceding pregnancy, non-health facility delivery of the preceding birth, being an in-migrant resident, multi-parity and being married.

**Conclusion:**

Generally, one in every two inter-birth intervals among 15–49 year-old women in Rufiji district is poorly spaced, with significant variations by socio-demographic and behavioral characteristics of mothers and newborns. Maternal, newborn and child health services should be improved with a special emphasis on community- and health facility-based optimum birth spacing education in order to enhance health outcomes of mothers and their babies, especially in rural settings.

## Background

Poorly spaced pregnancies have been documented worldwide to result in adverse maternal and child health outcomes [1]. An estimated 11 million children aged less than five years die yearly, with 99 percent of the deaths occurring in developing countries [2]. Evidence showing a relationship between shorter birth intervals and high infant and child mortality has been established globally [3-9]. In addition, extant evidence shows that closely spaced pregnancies are linked to low birth weight, intrauterine growth retardation, preterm delivery [10,11] and infant mortality [3]. Longer intervals have been proven to reduce fertility and consequently result in beneficial effects on population size [12].

With regard to maternal health, women who space their pregnancies inappropriately have an elevated risk of preeclampsia, high blood pressure, and premature rupture of membranes [1]. Undesirable consequences of shorter inter-birth intervals include perinatal, infant and child mortality and maternal mortality, and have been attributed to Maternal depletion syndrome, a biological phenomenon that refers to an inadequate recuperation of the mother from one pregnancy that avails an inhospitable intrauterine environment to accommodate a subsequent pregnancy [13,14].

Sibling competition has been reported to occur in the situation of shorter inter-birth intervals. With the birth of a child, a family may invest more of its limited resources in the care of the newborn while other children may receive an inadequate share of resources distributed among siblings. Furthermore, disease transmission is another mechanism through which shorter inter-birth intervals may be pernicious. Presence of numerous young children in a household may facilitate the spread of various communicable diseases such as respiratory infections and measles [15].

Research findings show also that births occurring within 2 years are riskier and their intervals are considered to be too short [16]. Recent findings show that intervals of 3 to 5 years are safer for both mother and infant compared to ≤ 2 years [17-19]. However, too long inter-birth intervals (>5 years) are associated with increased risk of complications such as preeclampsia because the mother loses protective effect from previous pregnancy [17].

In Tanzania like in many other African countries, fertility, maternal and child mortality are still high. Maternal mortality is estimated at 454 deaths per 100,000 live births, while neonatal mortality approximates 26 deaths per 1,000 live births [20]. While there seems to be a declining trend in the national total fertility rate (TFR), the figures remain high (national, 5.4 and Rufiji district, 5.1). Contraceptive prevalence of 34% nationally and 42% for the Coast region [20] in which Rufiji district is located, suggests the need to scale up efforts to increase access to family planning services. Therefore, analysis of birth intervals is of importance to the public because this stands a better chance to unveil possible circumstances leading to the inappropriate birth spacing, which, if acted upon, may beneficially affect fertility, child and maternal health.

In 2005, the World Health Organization (WHO) held a Technical Consultation and Scientific Review of Birth Spacing [21], and endorsed among other things that (1) after a live birth, the recommended interval before attempting the next pregnancy (i.e. birth-to-pregnancy interval) is at least 24 months in order to reduce the risk of adverse maternal, perinatal and infant outcomes and (2) after a miscarriage or induced abortion, the recommended minimum interval to next pregnancy is at least six months in order to reduce risks of adverse maternal and perinatal outcomes. Our analysis pertains to the first recommendation, which suggests that the minimum inter-birth or simply birth-to-birth interval should be 33 months (33 months = 24 months for not conceiving + 9 months period of pregnancy) in order to reduce adverse risks. This recommendation, according to WHO, was considered to be consistent with the WHO/UNICEF recommendation of breastfeeding for at least 2 years [21].

Considering slow progress towards achieving the Millennium Development Goals (MDGs) 4 (especially neonatal mortality) and 5 for Tanzania [22] and the limited evidence on birth spacing practices, this study attempts to (1) describe the median level of inter-birth interval (in months), (2) estimate proportions of inter-birth intervals below the recommended minimum inter-birth interval by characteristics of mother and child, and (3) identify factors associated with non-adherence to the recommended minimum inter-birth interval among multiparous women of childbearing age in Rufiji district of Tanzania.

## Methods

### Study area

The Rufiji Health and Demographic Surveillance System (HDSS) is located in Rufiji district of the Coast region, 178 kilometres south of Dar es Salaam, Tanzania. A HDSS is a longitudinal, population-based health and vital events registration system that monitors demographic events such as births, deaths, pregnancies, in- and out-migrations and socio-economic status of a geographically well-definedsetting of individuals, households and residential units. The Rufiji HDSS was incepted in September 1998 from the Tanzania Essential Health Interventions Project (TEHIP) and as of 2010, it was made up of 33 villages with over 16,000 households in which more than 80,000 people resided. The area is mainly rural with a scattered population, though clustering around Ikwiriri, Kibiti and Bungu townships is known. The largest and original native ethnic group in the HDSS is *Ndengereko.* Others include *Matumbi*, *Ngindo* and Z*aramo*. In terms of religion, about 90% of the people are Muslim. Most people speak their ethnic languages, even though the national language, *Kiswahili*, is well understood and widely spoken. Further details about the study area are available [23].

### Data and study population

This study is a secondary analysis of longitudinal data collected by the Ifakara Health Institute (IHI) in its Rufiji HDSS in Tanzania for a period of eleven years from 1^st^ January 1999 to 31^st^ December 2010. Access to the data was permitted by IHI, an institute that owns, manages and maintains the HDSS. The inception of the HDSS was approved by the Medical Research Coordinating Committee (MRCC) of the National Institute for Medical Research (NIMR) in Tanzania. This ethical approval is detailed elsewhere [24]. Data collection procedures of the HDSS require that every household is visited once every four months in order to update previously recorded household information and register new demographic events that may have occurred. Between household visits, community-based key informants report births and deaths as they occur. The Rufiji HDSS is an ongoing longitudinal population-based data generating platform.

A particular focus of the current study was on analyzing inter-birth intervals in light of the WHO’s recommendation on birth spacing. Therefore, resident women of the Rufiji HDSS aged 15–49 years who were followed-up for vital statistics, particularly birth history, were of interest. As the focus of this study was on closed inter-birth intervals, only women who had given birth at least twice (i.e. multiparous) were retained for this analysis. Those who had experienced adverse outcomes in any of their two consecutive births were very few and excluded in this analysis to be analyzed separately in light of the second recommendation of the WHO on birth spacing after experiencing an adverse outcome.

### Variables

This study examined inter-birth interval as a dependent (outcome) variable against background characteristics of the mother and the child. The inter-birth interval was collapsed into two categories according to the WHO recommendation: (1) <33 months, which was referred to as “non-adherence” or poor birth spacing, and (2) ≥33 months, referred to as “adherence” or appropriate birth spacing.

Independent variables investigated (with their categories in brackets) were (1) maternal age (broken into categories of 5 years interval size starting from 15–19 and ending with 45–49), (2) maternal education (secondary and higher, primary and never been to school), (3) maternal occupation (no job, self employment and formal employment), (4) marital status of the mother (married, single, ever married (i.e. divorced or widowed)), and (5) sex of the index child (female and male). Others were (6) place of residence (urban and rural), (7) number of births of the preceding pregnancy (singleton and multiple), (8) parity (2, 3 and ≥4), (9) place of delivery of the index pregnancy (health facility and elsewhere) and (10) HDSS entry type (enumeration and in-migration). During the start of the Rufiji HDSS, entry type of all people present at that time was enumeration. Entry into the HDSS area was also possible through birth or in-migration (migrating into the study area). No one of those who became members by birth was eligible for the current analysis because all were below 15 years of age throughout the follow-up period. Therefore the variable, HDSS entry type, had two categories only as enumeration and in-migration.

### Statistical analyses

An inter-birth interval was defined as a period of time (in months) between two consecutive live births [20]. This suggested that a woman could have several inter-birth intervals depending on her parity. Thus, the inter-birth intervals were calculated as

(1) $$I_{n}=\frac{D_{n}-D_{n-1}}{30.4},n=1,2,3..,k$$

Where I_n_ = n^th^ interval length between two consecutive births.

k = highest parity a woman has had at a given point in her reproductive lifetime,

*D*_*n*_ = date of birth of an n^th^ pregnancy,

D_*n-1 *_= date of birth of the preceding ((n-1)^th^) pregnancy and

30.4 = average number of days in a month

During data analysis, the inter-birth intervals were first analyzed descriptively in order to assess their distributional features. Then a binary outcome variable was defined by assigning the inter-birth intervals into one of the two categories according to the WHO recommendation such that

(2) $$\text{Non-adherence}=\{\begin{array}{l} 1\;\text{if}\;\text{an}\;\text{inter–birth}\;\text{interval}\;\text{<}\;33\;\text{months}\\ 0\;\text{if}\;\text{an}\;\text{inter–birth}\;\text{interval}\;\geq \;33\;\text{months} \end{array}$$

Proportions of the inter-birth intervals which were below the WHO recommendation by each of the independent variables were computed and presented, and the degree of association between them was tested using Chi-square (χ^2^). Factors associated with non-adherence were assessed using a multilevel logistic model in order to account for the fact that inter-birth intervals of the same woman are highly correlated. The intervals were considered to be nested or clustered among women. This procedure was conducted using the STATA command, ‘xtlogit’, to obtain random-effects logistic regression results. Odds ratios (OR), their corresponding 95% confidence intervals (CI) and P-values were calculated and presented as well. In interpreting effects such as OR, confidence intervals among other things play the role of P-values. Therefore, presenting OR and their corresponding confidence intervals without the P-values may suffice. However, we also presented the P-values because some readers prefer them for quick inferences about significance. The whole process of data analysis was conducted using STATA (version 11) statistical software (StataCorp, Texas, USA). A cut-off point (significance level) at which a factor was identified as a predictor of the outcome, non-adherence, was 5%.

## Results

**Figure 1:**
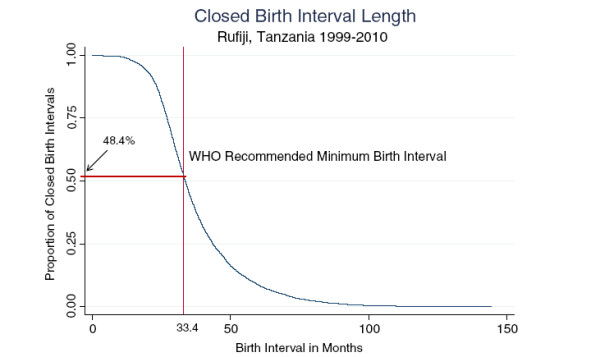
Birth spacing levels among 15–49 year-old women in Rufiji, Tanzania, 1999–2010 (n = 15,373).

From 1^st^ January 1999 to 31^st^ December 2010, a total of 15,373 closed inter-birth intervals were recorded from 8,980 women aged 15–49 years in the Rufiji HDSS. The median inter-birth interval was 33.4 months (inter-quartile range = 16.5). Of these 15,373 intervals, 48.4% (n = 7,446) were below the WHO recommended minimum inter-birth interval of 33 months between two consecutive live births for better maternal and child health outcomes (Figure 1). These inappropriate inter-birth intervals were observed among 40.9% (3,668) of all the women followed. On average, each of the women who spaced any of her births poorly had about 2 non-adherent inter-birth intervals.

### Inter-birth intervals by background characteristics

**Figure 2:**
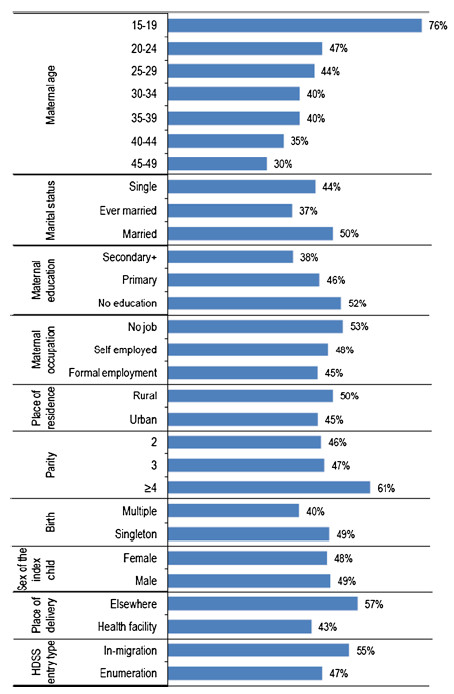
Percent distribution of inter-birth intervals less than 33 months long between two consecutive live births by characteristics of mother and child in Rufiji, Tanzania, 1999–2010 (n = 15,373).

Figure 2 presents bivariate analysis of the inter-birth intervals by background characteristics of the mother and the child. The results show that maternal age was inversely related with non-adherence to the recommended minimum interval between two consecutive live births. The proportion of the inter-birth intervals that were non-adherent was highest (76%) among youngest (15–19) women and declined rapidly in subsequent age categories to as low as 30% among the oldest (45–49) women (P<0.001). In terms of marital status, the highest proportion (50%) of non-adherent inter-birth intervals was observed among married women and the lowest (37%) occurred among ever married (divorced or widowed) women (P<0.001). Furthermore, we found that the lower the maternal education the higher the proportion of non-adherence. The data showed that 52%, 46% and 38% of the inter-birth intervals that were non-adherent occurred among women with no education, primary education and secondary and higher education respectively (P<0.001). Likewise, non-adherence was 53% among women who had no job, dropped to 48% among women who were self-employed and lowest (45%) among women with formal employment (P = 0.058). Regarding place of residence, non-adherence was higher among rural resident women than their urban counterparts (50% versus 45%) (P<0.001). Moreover, parity of at least four children was associated with the highest proportion (61%) of non-adherence (P<0.001).

On the other hand, we found that 49% of the inter-birth intervals beginning with a singleton birth were non-adherent. This proportion was 40% for inter-birth intervals beginning with multiple births (i.e. twins triplet etc.) and the difference was statistically significant (P = 0.002). While 43% of the inter-birth intervals beginning with children born in health facilities were non-adherent, 57% of the inter-birth intervals beginning with births that did not occur in health facilities were non-adherent (P<0.001).

We also observed a significantly higher proportion of non-adherence among women who became members of the study area through in-migration compared to women who became members through enumeration (55% versus 47%) (P<0.001). Finally, the proportion of non-adherence was similar between inter-birth intervals beginning with male and those beginning with female children (48% versus 49%) (P = 0.215).

### Correlates of non-adherence

Results of the multivariate (adjusted) random-effects logistic regression of the factors associated with non-adherence to the WHO recommended minimum inter-birth intervals are presented in Table 1. Findings reveal that the lower the maternal age the higher the likelihood of non-adherence and vice versa. Inter-birth intervals observed among women aged between 15–19 years were about 14 times more likely to be poorly spaced compared to inter-birth intervals observed among women aged 45–49 (OR = 13.65, 95% CI 9.63-19.35). This trend continued, but with a declining magnitude of the odds ratios for subsequent age categories, except the age category 40–44 in which the likelihood of non-adherence was low and not different from that for the 45–49 age category (OR = 1.31, 95% CI 0.95-1.83). In terms of marital status, the likelihood that an inter-birth interval was less than 33 months was 44% and 36% less likely among ever married women (OR = 0.56, 95% CI 0.48-0.66) and single women (OR = 0.64, 95% CI 0.57-0.73) respectively compared to married women. On the other hand, inter-birth intervals observed among women who had no formal education (never been to school) were 27% more likely to be non-adherent to the recommendation compared to inter-birth intervals observed among women who had secondary education and higher (OR = 1.27, 95% CI 1.01-1.60). Non-adherence to the recommendation was also significantly associated with increasing parity of the mother (Para 3: OR = 1.29, 95% CI 1.19-1.40; Para ≥4: OR = 2.54, 95% CI 2.28-2.85).

**Table 1:** Random-effects logistic regression of factors associated with poory spaced inter-birth intervals (<33 months) in Rufiji, Tanzania: 1999–2010 (n = 15,158)

**Variable**	**Odds ratio (OR)**	**95% Confidence Interval (CI)**	**P-Value**
**Maternal age (years)**			
15-19	13.65	9.63-19.35	<0.001
20-24	4.30	3.16-5.86	<0.001
25-29	2.40	1.77-3.26	<0.001
30-34	2.07	1.52-2.80	<0.001
35-39	1.64	1.21-2.24	0.002
40-44	1.31	0.95-1.83	0.100
45-49 (ref.)	1.00	––	––
**Marital status of mother**			
Married (ref.)	1.00	––	––
Ever married (widowed or divorced)	0.56	0.48-0.66	<0.001
Single	0.64	0.57-0.73	<0.001
**Maternal education**			
Secondary/higher (ref.)	1.00	––	––
Primary	1.09	0.87-1.37	0.456
Never been to school	1.27	1.01-1.60	0.042
**Place of residence**			
Urban (ref.)	1.00	––	––
Rural	1.04	0.95-1.13	0.400
**Parity**			
2 (ref.)	1.00	––	––
3	1.29	1.19-1.40	<0.001
≥4	2.54	2.28-2.85	<0.001
**Birth**			
Singleton (ref.)	1.00	––	––
Multiple	0.74	0.57-0.96	0.023
**Place of delivery of the index child**			
Health facility (ref.)	1.00	––	––
Elsewhere	1.85	1.71-2.00	<0.001
**HDSS entry type**			
Enumeration (ref.)	1.00	––	––
In-migration	1.32	1.21-1.45	<0.001

Model characteristics.

/lnsig2u = −1.43 (−1.92 to −0.95); sigma_u = 0.49 (0.38 to 0.62); rho = 0.07 (0.04 to 0.11). Number of groups = 8,807; Observations per group: min = 1, average = 1.7, maximum = 6. ref. = reference/baseline category; HDSS = Health and Demographic Surveillance System.

Furthermore, inter-birth intervals beginning with multiple births were 26% less likely to be non-adherent compared to those beginning with singleton births (OR = 0.74, 95% CI 0.57-0.96). Also inter-birth intervals beginning with children born elsewhere other than in health facilities were 85% more likely to be non-adherent compared to those beginning with children born in health facilities (OR = 1.85, 95% CI 1.71-2.00). Finally, inter-birth intervals observed among women who became members of the HDSS through in-migration were 32% more likely to be non-adherent compared to those from women who became members of the HDSS through enumeration (OR = 1.32, 95% CI 1.21-1.45).

Having adjusted for all variables in the multivariate model, the association between non-adherence to the recommended minimum inter-birth interval and place of residence was not significant (OR = 1.03, 95% CI 0.95-1.12).

## Discussion

Our findings reveal that close to half of the inter-birth intervals in Rufiji district were below the WHO recommended minimum of 33 months between two consecutive live births for better maternal and child health outcomes. This corresponded to more than two in every five women (results not shown) not adhering to the recommendation, implying that a significant proportion of mothers in the study area may be at risk of adverse maternal and newborn health outcomes due to improper birth spacing. The median inter-birth interval of 33.4 months observed in this study is similar to that reported in the recent Tanzania Demographic and Health Survey (TDHS) [20] and that of Ethiopia, one of the countries with the highest fertility rate in Africa [25].

The study further identified a number of factors associated with non-adherence to the minimum recommendation of the WHO on birth spacing. Maternal age and non-adherence were inversely related, such that the younger the maternal age the higher the likelihood of non-adherence to the recommendation and vice versa. We found also that the higher the parity the higher the likelihood of non-adherence. However, there was no statistical interaction between maternal age and parity in the prediction of non-adherence to the recommended minimum inter-birth interval. It is possible that older women may have already achieved their desired family sizes as age advances compared to younger women, hence likely to delay subsequent births [16]. Older women may also be less fertile compared to the younger ones, a situation that reduces their probability of conception thus leading to longer inter-birth intervals [16,26]. Our findings are consistent with several others which have similarly shown that older mothers tend to have longer birth intervals [27,28].

Ever married women (divorced or widowed) and those who were still single were less likely to space their births poorly compared to married women. This suggested that women who are currently not married may have less opportunity for childbearing and consequently likely to have longer inter-birth intervals than their married counterparts. Research shows that frequency of sexual intercourse tends to be higher in marriage than in any other category of marital status [29,30], thus an increased chance of conception and eventually births. On the other hand, low utilization of contraceptives in Tanzania especially among married women [20] might be a contributing factor to the higher likelihood of non-adherence to the recommendation among married women. This observation agrees with that reported in Asia [5]. Additionally, it is possible that unmarried women may be younger or perhaps still in schools thus likely to have had their pregnancies mistakenly. Consequently, they may be unprepared for childrearing and their inter-birth intervals may thus be longer.

Absence of formal education (never been to school) was associated with higher likelihood of non-adherence to the recommended minimum inter-birth interval. Although some studies such as one conducted in Korea in the 1980s showed that better educated women space births poorly [31], our findings are consistent with recent findings [28,32]. This may be partly attributable to the transformational role that education plays as a catalyst for change that informs and influences decisions and choices [33,34]. While the contemporary literature in the field seem to agree about the relationship between maternal education and birth spacing, a reason for the diversity remains less clear.

Inter-birth intervals beginning with multiple births were less likely to be non-adherent than those beginning with a singleton birth. This may be partly due to double or even more logistical difficulties and financial expenses that the family has to incur in the process of upbringing two or more children simultaneously. It may also be due to parents’ satisfaction as far as their desired number of children or family size is concerned, thus likely to delay the next birth. For example, a qualitative study with health service providers in Egypt reports that “… postponing pregnancy for longer periods usually occurs following the second child …” [35], which may also be the case when one pregnancy results into multiple births.

With respect to place of delivery, inter-birth intervals beginning with births that did not occur in health facilities were more likely than those beginning with births that occurred in health facilities to be non-adherent to the WHO recommendation. Women who seek antenatal care or delivery services from health facilities may have access to education on optimum birth spacing, breastfeeding, family planning and adverse risks of pregnancy and pregnancy outcome. This is thought to have a greater role in influencing pregnancy preparedness and care [35]. Therefore, it is important to promote and encourage health care seeking from health facilities during antenatal, childbirth, postnatal and throughout childrearing period.

Finally, evidence of in-migrant women being more likely to space their births poorly compared to native residents of the study area indicates the possibility of cultural differences, beliefs and practices of birth spacing [36]. Similarly, it may be that native residents of the study area were better informed on optimal birth spacing from different sources including the presence of the Rufiji HDSS in particular than the in-migrants, who may not have had a longer duration of exposure to the HDSS.

### Limitations

Unfortunately, there were no data available regarding duration of breastfeeding, contraceptive use, religion, biological and genetic factors, which, if available, would have alighted more on the question posed. There may have been a possibility of misclassifying an inter-birth interval ending in a preterm but with a live birth as inappropriately spaced. Since only one district was studied, our findings may not be generalized to the whole population of Tanzania.

## Conclusion

Close to a half of all inter-birth intervals in Rufiji district is poorly spaced. Younger maternal age, low maternal education, multiple births from the preceding pregnancy, non-health facility delivery of the preceding birth, being an in-migrant resident, multi-parity and being married are significantly associated with non-adherence to the WHO recommended minimum inter-birth interval of 33 months for better maternal and child health outcomes. Improving maternal, newborn and child health (MNCH) services with a special attention on birth spacing is important in Rufiji district. Community- and health facility-based optimum birth spacing education is urgently required to enhance birth spacing and consequently improve health outcomes of mothers and children in Tanzania.

## Competing interest

The authors declare that they have no competing interests.

## Authors’ contributions

AE conceptualized the research question and wrote the manuscript drafts. AE, KB, SM and AS designed the study and analyzed the data. DM, GM and HM participated in designing the study and critically reviewed the manuscript drafts. All authors read and approved the final draft of the manuscript.

## Pre-publication history

The pre-publication history for this paper can be accessed here:

http://www.biomedcentral.com/1471-2393/12/152/prepub
